# Optogenetic insights on the relationship between anxiety-related behaviors and social deficits

**DOI:** 10.3389/fnbeh.2014.00241

**Published:** 2014-07-16

**Authors:** Stephen A. Allsop, Caitlin M. Vander Weele, Romy Wichmann, Kay M. Tye

**Affiliations:** ^1^Department of Brain and Cognitive Sciences, Picower Institute for Learning and Memory, Massachusetts Institute of TechnologyCambridge, MA, USA; ^2^Harvard Medical School, Harvard UniversityBoston, MA, USA

**Keywords:** anxiety, social deficits, optogenetics, basolateral amygdala, ventral hippocampus, mouse models of affective disorders, social interaction, autism

## Abstract

Many psychiatric illnesses are characterized by deficits in the social domain. For example, there is a high rate of co-morbidity between autism spectrum disorders and anxiety disorders. However, the common neural circuit mechanisms by which social deficits and other psychiatric disease states, such as anxiety, are co-expressed remains unclear. Here, we review optogenetic investigations of neural circuits in animal models of anxiety-related behaviors and social behaviors and discuss the important role of the amygdala in mediating aspects of these behaviors. In particular, we focus on recent evidence that projections from the basolateral amygdala (BLA) to the ventral hippocampus (vHPC) modulate anxiety-related behaviors and also alter social interaction. Understanding how this circuit influences both social behavior and anxiety may provide a mechanistic explanation for the pathogenesis of social anxiety disorder, as well as the prevalence of patients co-diagnosed with autism spectrum disorders and anxiety disorders. Furthermore, elucidating how circuits that modulate social behavior also mediate other complex emotional states will lead to a better understanding of the underlying mechanisms by which social deficits are expressed in psychiatric disease.

## Social deficits in psychiatric disease

Social deficits have emerged as one of the major symptoms observed in many psychiatric diseases including schizophrenia, depression, anxiety, obsessive compulsive disorder, and Fragile X (Kennedy and Adolphs, 2012; American Psychiatric Association, 2013; Derntl and Habel, 2013). In addition, some diseases, such as autism and social anxiety disorder, are primarily characterized by deficits in the social domain (Stein and Stein, 2008; Losh et al., 2009; Kennedy and Adolphs, 2012). While impairments in social function are found in a variety of psychiatric disorders (Ormel et al., 1993; Wohlfarth et al., 1993; American Psychiatric Association, 2013), the prevalence of social anxiety disorder and the high rate of co-morbidity between anxiety and autism spectrum disorders highlights the need to understand the relationship between anxiety and social behavior (De Bruin et al., 2007; Simonoff et al., 2008; Stein and Stein, 2008). As a result, in this review we focus on anxiety and its link to deficits in social behaviors. For a more extensive discussion on how social function is affected in psychiatric disease see the review by Kennedy and Adolphs (2012).

Anxiety is characterized as a heightened state of arousal and vigilance that occurs in the absence of an immediate threat (Davis et al., 2009). Although it may have evolved as an adaptive behavioral state, anxiety can become pathological when it is no longer an appropriate response to a given situation (American Psychiatric Association, 2013). Anxiety can be conceptualized as having two components, state anxiety and trait anxiety. Whereas trait anxiety refers to an individual's personality and predisposition for anxiety, state anxiety refers to the emotional response generated by a perceived threat (Spielberger et al., 1983; Endler and Kocovski, 2001). Clinically, general anxiety disorder is characterized by excessive and uncontrollable anxiety as a result of non-threatening stimuli, accompanied by defined physiological symptoms that cause serious distress or impairment that must not be caused by another psychiatric or medical disorder (Fricchione, 2004; Hoge et al., 2012; American Psychiatric Association, 2013). Although it is implicit in the diagnosis of anxiety disorders that patients likely have an increase in state and trait anxiety, this is not usually directly assessed. As a result, in this review we refrain from using those terms when discussing clinical findings.

In patients with general anxiety, social function is significantly affected and has been found to be an important cause for disability when comparing anxious patients to controls (Schonfeld et al., 1997; Kessler et al., 1999; Kroenke et al., 2007). In young adults with anxiety, these deficits may be even more detrimental because they occur during a period vital for social development (Wittchen et al., 1998). Aside from the impairments and disability in the social domain that occur with generalized anxiety, anxiety itself can be limited to social functioning—which is exemplified in social anxiety disorder. To be diagnosed with social anxiety disorder, a patient must suffer from significant distress or impairment that interferes with ordinary routine in social settings, at work or school, or during everyday activities (American Psychiatric Association, 2013). Individuals with social anxiety disorder avoid interpersonal interactions whenever possible. If they must endure one, it is with extreme emotional and physical discomfort (Schneier, 2006; Stein and Stein, 2008).

The lack of specific pharmacological treatments for neuropsychiatric diseases such as social anxiety disorder and autism points to a need for a greater understanding of the neural mechanisms that mediate social behaviors and how they are affected by anxiety-related illnesses. Current pharmacological treatment approaches for social anxiety disorder and autism spectrum disorders utilize drugs which are also used to treat other psychiatric disorders (e.g., anxiety and depression) (Gordon et al., 1993; Stein et al., 1998; Fedoroff and Taylor, 2001; Malone et al., 2002; Rodebaugh et al., 2004). In addition, treatments for autism are often ineffective at treating social pathologies (McDougle et al., 2005; but see Hollander et al., 2007; Andari et al., 2010). These data call for a better understanding of the neural correlates underlying these disorders.

## Optogenetics and the use of animal models to study psychiatric disease

Experimental approaches in human subjects have yielded significant insights about brain regions involved in anxiety (Etkin and Wager, 2007; Ressler and Mayberg, 2007) and social behavior (Adolphs, 2003; Lieberman, 2007). However, there are considerable ethical and technological limitations to using humans as experimental subjects [Council for International Organizations of Medical Sciences, 2002; Institute of Medicine (US) Forum on Neuroscience and Nervous System, 2008]. Establishing causal relationships between specific neuropsychiatric symptoms and precise brain mechanisms requires invasive techniques that are not suitable for human subjects. In addition, the expense of drug development for psychiatric disorders dictates that drug targets are validated in more economical systems prior to being tested in humans (Frantz, 2004).

Animal models are one important means to address the limitations of human neuroscience research (Cryan and Holmes, 2005; Nestler and Hyman, 2010). Animal models enable more invasive methodologies and the application of new technologies in order to provide information about the basic mechanisms involved in driving behavior (Nestler and Hyman, 2010; Aston-Jones and Deisseroth, 2013; Cruz et al., 2013; Kim et al., 2013b). One such technology is optogenetics. Optogenetics involves the integration of light-sensitive proteins, called “opsins,” into cell membranes allowing for millisecond temporal control of cellular activity by photostimulation (Boyden et al., 2005; Fenno et al., 2011). The most commonly used light-sensitive opsins are channelrhodopsins (ChRs), halorhodopsins (NpHRs), and Archaerhodopsins (Archs) (Soliman and Trüper, 1982; Mukohata et al., 1988; Nagel et al., 2002, 2003; Zhang et al., 2007a,b). ChRs are a class of cation channels that, when exposed to blue light, cause the depolarization of neuronal membranes where opsins are expressed and results in neuronal excitability (Nagel et al., 2003; Boyden et al., 2005). In contrast, NpHRs are chloride pumps and Archs are proton pumps that, when exposed to yellow light, cause the hyperpolarization of neuronal membranes and results in subsequent inhibition (Zhang et al., 2007a,b; Chow et al., 2010; Gradinaru et al., 2010). Through various targeting strategies, optogenetics allows a high level of spatial and temporal control of specific, molecularly defined neuronal circuits (Tye and Deisseroth, 2012). Importantly, optogenetics has been successfully used to elucidate neuronal circuits involved in many complex behaviors relevant to rodent models of psychiatric disease (Nieh et al., 2013; Deisseroth, 2014).

However, whether it is possible to model psychiatric disease in animals is controversial. For instance, some diagnostic features of psychiatric diseases include terms such as sadness, guilt, delusions, and disorganized thinking (American Psychiatric Association, 2013). These symptoms are difficult to ascertain in animal models. In addition, the variability in clinical presentation of psychiatric diseases makes modeling emotional disease states in animals a challenge. Nevertheless, scientists have been able to successfully create models that recapitulate important features of various psychiatric diseases such as anxiety (Lister, 1990; Lang et al., 2000), depression (Willner, 1984; Castagné et al., 2001), and autism (Lewis et al., 2007; Ting and Feng, 2011).

## Using animal models to understand anxiety and social behavior

Neuroscientists have made significant strides toward understanding the neural mechanisms of anxiety (Shin and Liberzon, 2009; Dias et al., 2013). Rodent models of anxiety have been a useful tool in this regard as they have been shown to have both face validity and predictive validity (Lister, 1990; Cryan and Holmes, 2005) and have led to mechanistic and potential therapeutic insights (Cryan et al., 2003; Holmes et al., 2003; Rudolph and Möhler, 2004). In order to study anxiety-like behaviors in rodents, a variety of tests and strategies have been employed. The three main strategies involve ethological or conflict-based tests, classical conditioning tests, and genetic models (Lister, 1990; Cryan and Holmes, 2005). Ethological/conflict-based tests rely on unconditioned responses based on innate behaviors while classical conditioning tests rely on learned responses to experimental conditions. Finally, genetic models rely on specific gene manipulations leading to different levels of “trait” anxiety. This has led to the discovery of a number of promising cellular mechanisms involved in anxiety-related behaviors (Kent et al., 2002; Wu et al., 2008; Johansen et al., 2011). However, it should be noted that in rodents, the tests used to assess “trait” anxiety are the same ones utilized to assess “state” anxiety [i.e., elevated plus maze (EPM), open field test (OFT)], thus it is difficult to dissociate whether genetic mouse models have truly increased trait anxiety or excessive state anxiety to the tested context (Lister, 1990; Cryan and Holmes, 2005).

Thorough explanations of the tests used to study anxiety-like behaviors in rodents have been presented in previous literature (Lister, 1990; Rodgers, 1997; Finn et al., 2003; Fuchs and Flugge, 2006; Cryan and Sweeney, 2011; Campos et al., 2013; Kumar et al., 2013). As such, we will not discuss them in detail here (For an overview of the most commonly used tests, see Table 1).

**Table 1:**
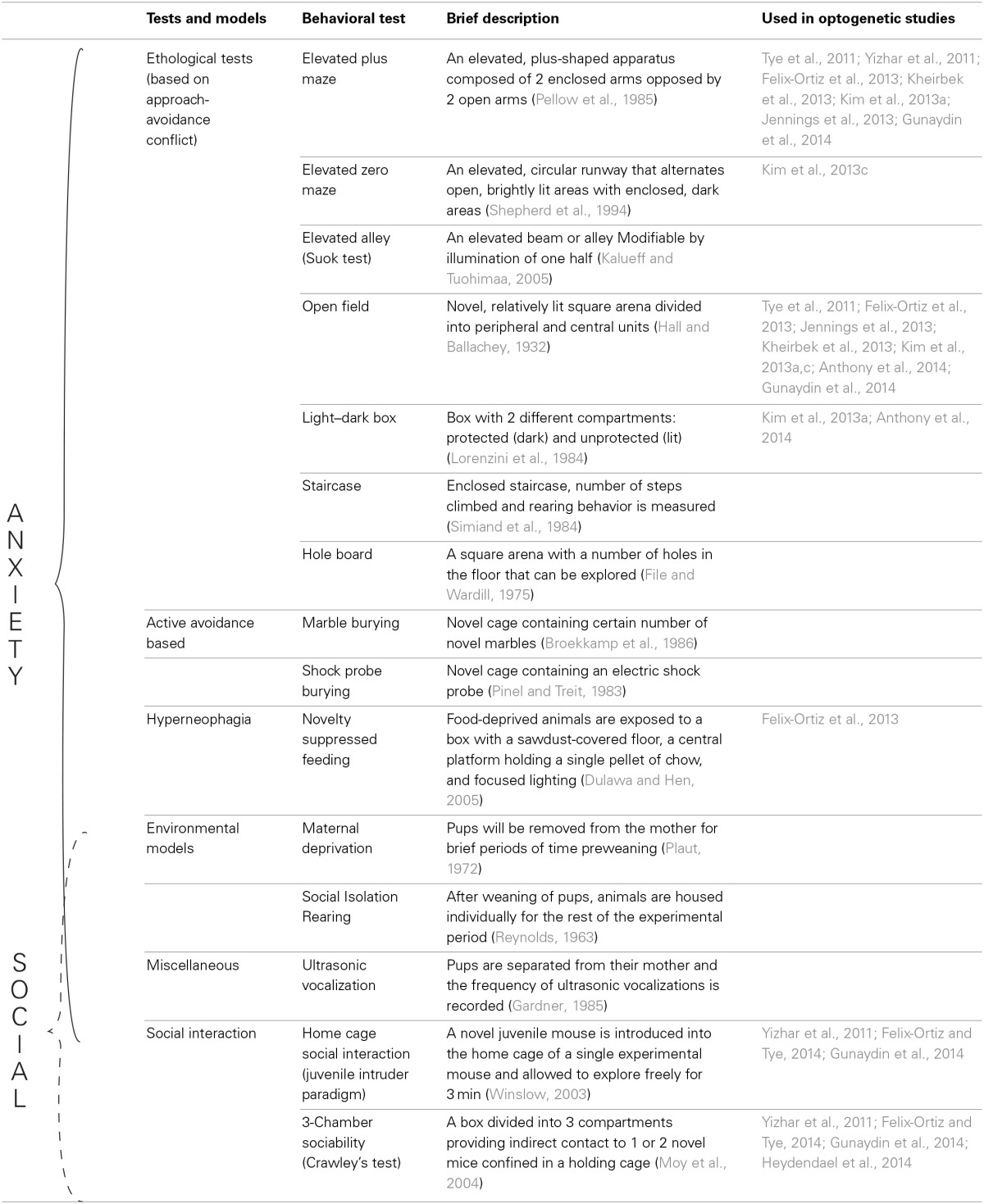
**Tests used to assess anxiety as well as social behavior in rodents**.

Table focuses on non-aggressive, non-mating social assays.

**Figure 1:**
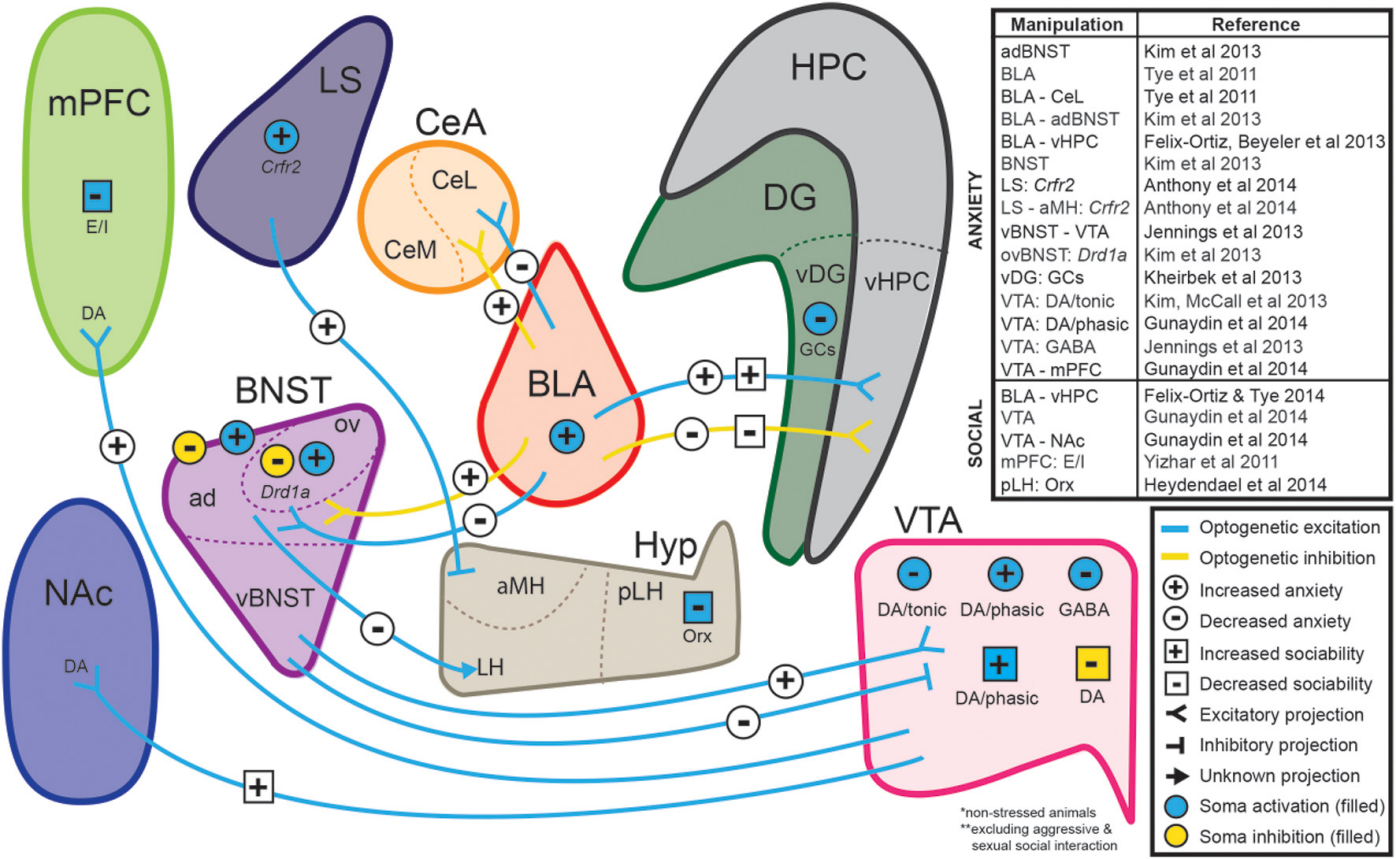
**Neural circuits implicated in anxiety and social function through optogenetic investigations**. The ability to selectively manipulate distinct neuronal populations and projections with high temporal resolution is a significant advantage of optogenetic approaches. Several recent studies have used optogenetic strategies to establish causal relationships between specific neuronal projections and behaviors relevant to anxiety and sociability in non-stressed animals. Abbreviations: ad, anterodorsal subdivision of the bed nucleus of the stria terminalis; aMH, anterior hypothalamic area of the medial hypothalamus; CeA, central amygdala; *Crfr2,* type-2 corticotropin-releasing factor receptor; BLA, basolateral amygdala; BNST, bed nucleus of the stria terminalis; DA, dopaminergic neurons; DG, dentate gyrus; *Drd1a, dopamine receptor 1a;* E/I, manipulation of excitatory/inhibitory balance; GABA, GABAergic neurons; GCs, granule cells; HPC, hippocampus; Hyp, hypothalamus; LH, lateral hypothalamus; LS, lateral septum; mPFC, medial prefrontal cortex; NAc, nucleus accumbens; Orx, orexin neurons; ov, oval nucleus of the bed nucleus of the stria terminalis; pLH, posterior lateral hypothalamus; vBNST, ventral subdivision of the bed nucleus of the stria terminalis; vDG, ventral dentate gyrus; vHPC, ventral hippocampus; VTA, ventral tegmental area. (For review of optogenetic investigations into the neural circuitry involved in aggression and sexual behavior, we refer readers to Anderson, 2012).

The combination of these well-established tests with recent advances in optogenetics (Fenno et al., 2011; Tye and Deisseroth, 2012; Deisseroth, 2014) has highlighted the importance of combining these established behavioral models with new technology to uncover the mechanistic basis of anxiety disorders. In fact, various groups have employed tests such as the EPM and OFT to highlight the causal role of various circuits in modulating anxiety-like behaviors in mice (Tye et al., 2011; Yizhar et al., 2011; Felix-Ortiz et al., 2013; Jennings et al., 2013; Kim et al., 2013a,c; Kheirbek et al., 2013; Anthony et al., 2014; Heydendael et al., 2014; Gunaydin et al., 2014) (see Figure 1). In these assays, the temporal precision of optogenetics is underscored by allowing for within-subject and within-session comparisons.

Just as in anxiety, animal models have also been a useful tool for scientific inquiry into the brain regions, connections, and signaling involved in social function (Cacioppo, 2002; Insel and Fernald, 2004; Crawley, 2007; Adolphs, 2009; Silverman et al., 2010). Many animals are known to display a wide array of social behaviors that can be assayed in a laboratory setting (Hau et al., 2002). For example, *Caenorhabditis elegans* and *Drosophila melanogaster* have been successfully used to study the genetic basis of social behaviors such as aggregation, mating, and aggression (Antony and Jallon, 1982; Liu and Sternberg, 1995; Lee and Hall, 2000; Srinivasan et al., 2008; Macosko et al., 2009). For a synopsis of insights provided by the rich genetic toolkits of these model organisms, refer to the review by Sokolowski (2010).

Various studies have utilized the social behaviors found in rodents to find neural substrates of innate behaviors like aggression and mating (Choi et al., 2005; Lin et al., 2011; Anderson, 2012). Others have made strides in understanding the basis of behaviors such as emotional contagion, empathic responses, and observational learning in rodents (Jeon et al., 2010; Atsak et al., 2011; Bartal et al., 2011). Social behavior has also been studied extensively in non-human primates (Brown and Schafer, 1888; De Waal and Suchak, 2010). Primates exhibit a very complex set of social behaviors including the formation of long-term alliances and “friendships” that lead to social interactions and hierarchies that closely resemble human social structures (Cheney et al., 1986; Whiten et al., 1999; Adolphs, 2009).

Another important animal model for studying social behavior is the prairie vole. Prairie voles maintain long-term social attachments after mating, known as a pair bond (Getz et al., 1981; Carter et al., 1995; Wang and Aragona, 2004; Young and Wang, 2004) and thus serve as an appropriate analog to the type of social bonds observed in humans (Cacioppo, 2002; Insel and Fernald, 2004; Adolphs, 2009). To date, anatomical and pharmacological techniques have been used in combination with behavioral assays of pair bonding in prairie voles to reveal the importance of oxytocin, vasopressin, dopamine, and opioids in selective social attachment (Insel and Hulihan, 1995; Cho et al., 1999; Aragona et al., 2003, 2006; Resendez et al., 2012).

Just as with anxiety, optogenetics offers a great opportunity to begin elucidating the circuits involved in social behavior. Various optogenetic manipulations have provided recent evidence about the neural basis for a number of different rodent social behaviors (Gunaydin et al., 2014; Reviewed by Anderson, 2012; Yizhar, 2012) and application of optogenetic approaches to models such as the prairie vole holds great promise for future insight into the neurobiology of social attachments and behavior.

## Experimental/behavioral evidence of a correlation between general anxiety and social dysfunction

From clinical data, there seems to be a significant link between anxiety and impaired social interaction. Considering the body of experimental evidence on anxiety and social interaction, there is compelling evidence to support a correlation between anxiety and impaired social interaction [Although there is a wide range of social animal behavior, here we focus on non-aggressive, non-sexual, reciprocal social interaction (For overview see Table 1)]. This relationship may provide clues for understanding the mechanism by which co-expression of anxiety and social deficits arise.

Human studies have shown that anxiety disorders are common in individuals with autism. For example, in a side-by-side comparison, children with high-functioning autism have higher levels of reported anxiety than controls without autism (Muris et al., 1998; Gillott et al., 2001; MacNeil et al., 2009; White et al., 2009). Likewise, youth with anxiety disorders have higher scores on the autism spectrum disorder symptom scale than healthy controls (Pine et al., 2008). In patients with Williams Syndrome, social dysfunction increases as anxiety levels increase (Riby, 2013). Further, pharmacological studies have shown that benzodiazepines, commonly prescribed for generalized anxiety, have also been used as treatments for social anxiety disorder (Davidson et al., 1993; Jefferson, 2001; Schneier, 2006). Additionally, selective serotonin reuptake inhibitors (SSRIs), which increase serotonin concentration, have been shown to have enhancing effects on complex social behaviors (Knutson et al., 1998; Harmer, 2002) and have been used for treating general anxiety disorder as well as social anxiety disorder (Stein et al., 1998; Van der Linden et al., 2000; Rickels et al., 2003). Lastly, oxytocin, a neuropeptide well-known for its role in enhancing social function (Domes et al., 2007; Guastella et al., 2008; Meyer-Lindenberg et al., 2011), also carries anxiolytic properties (Labuschagne et al., 2010; Missig et al., 2010). These studies suggest there may be some common neural mechanisms underlying anxiety and social behavior in humans.

Animal studies also support the same relationship between anxiety and impaired social function. Early rodent studies showed that anxiolytics prevent decreases in social interaction that occur when animals are placed in anxiogenic environments (i.e., novel environments, bright light) (File et al., 1976; File and Hyde, 1978; File and Seth, 2003). Further, various autistic mouse models exhibit both pronounced deficits in sociability as well as enhanced anxiety-like behaviors (Nakatani et al., 2009; Silverman et al., 2010; Peça et al., 2011). In line with human literature, serotonin activity in rodents mediates social behaviors such as aggression and social reward (Saudou et al., 1994; Dölen et al., 2013), while reduced serotonin signaling increases anxiety (Heisler et al., 1998; Ramboz et al., 1998; Gross et al., 2002; Akimova et al., 2009). Lastly, oxytocin appears to facilitate social behaviors (Donaldson and Young, 2008; Insel, 2010; Lukas et al., 2011; Meyer-Lindenberg et al., 2011) and attenuate anxiety-related behaviors in rodents (Amico et al., 2004; Insel, 2010; Viviani et al., 2011; Knobloch et al., 2012) (See Table 2 for summary). These studies further demonstrate that anxiety and social function are intimately linked and suggest that pathologies in the respective domains may share a common neural mechanism.

**Table 2:**
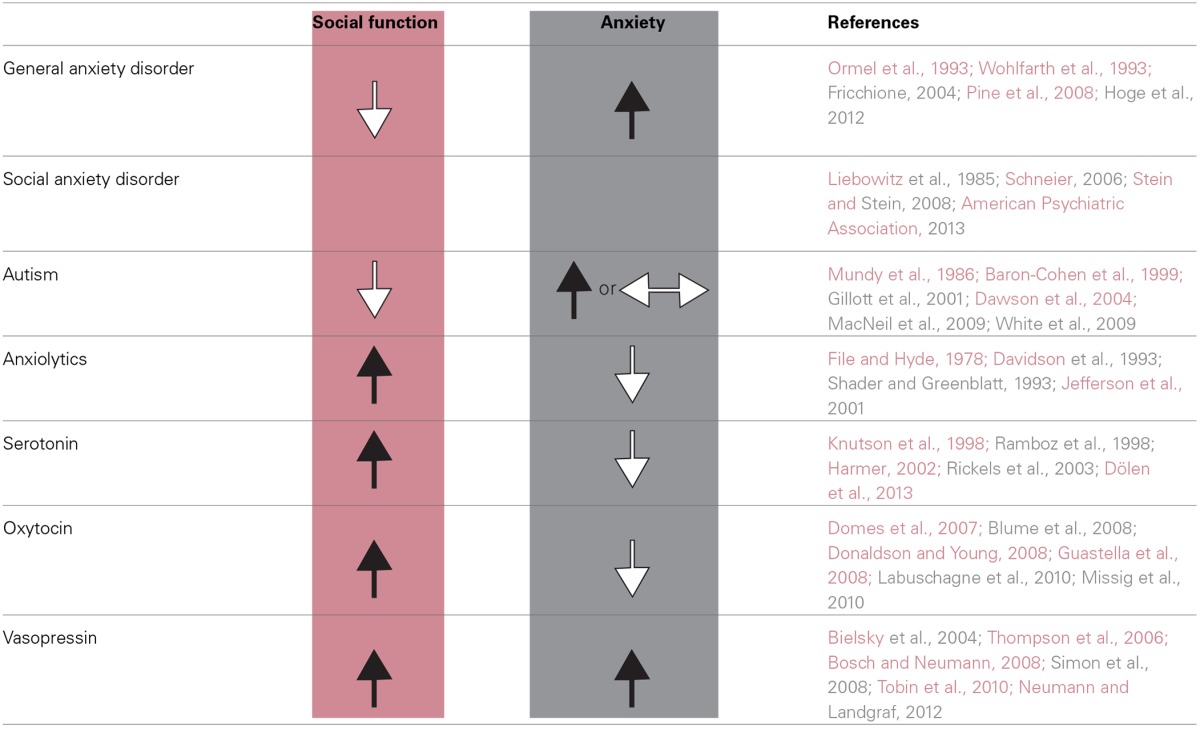
**Human and animal data supports a correlation between social dysfunction and anxiety**.

Experimental manipulations and diseases that lead to increases in anxiety tend to also lead to decreases in social function. Similarly, conditions that lead to increases in social function tend to also lead to anxiolysis. A notable exception to this trend is Vasopressin. References are color coded to indicate whether they refer to social function (pink), anxiety (gray), or both.

Although there is evidence that increased anxiety is correlated with deficits in social function, there are several caveats. For example, in Williams syndrome, there is a tendency to engage in “pro-social,” friendly behavior despite these patients exhibiting increased anxiety (Porter et al., 2009; Stinton et al., 2010; Riby et al., 2013). This finding may be explained by observations that although patients afflicted with Williams syndrome appear to be highly social, they often have abnormal social cognition that can lead to serious deficits in appropriate social interaction (Bellugi et al., 2000; Meyer-Lindenberg et al., 2005; Riby et al., 2013). Another finding at odds with the trend discussed above is that the neuropeptide vasopressin, well-known for its positive influence on social behavior, has also been shown to be anxiogenic in both humans and rodents (Bielsky et al., 2004; Thompson et al., 2006; Bosch and Neumann, 2008; Donaldson and Young, 2008; Simon et al., 2008; Tobin et al., 2010; Neumann and Landgraf, 2012). Lastly, some autistic mouse models contain deficits in social interaction but show no changes in anxiety-like behaviors (McFarlane et al., 2008; Liu and Smith, 2009). These are a few examples that highlight the fact that although anxiety and social dysfunction are often co-expressed, there are exceptions. Importantly, the expression of behaviors relevant to anxiety and social interaction encompasses a wide array of behaviors. Therefore, it is possible that certain subsets of these behaviors share the same neural machinery, while others may not.

## The amygdala and its role in anxiety and social behavior

We can begin to understand the connection between anxiety and social behavior by identifying neural substrates that mediate both behaviors. The amygdala is one such region. Initial work elucidating the amygdala's role in fear and anxiety was the observation that bilateral destruction of the amygdala results in attenuated fear (Klüver and Bucy, 1939). Since then, human studies have consistently shown that the amygdala is involved in processing emotional faces, particularly those involving fearful or threatening expressions (Adolphs et al., 1994; Morris et al., 1996; Rauch et al., 2003; Etkin et al., 2004). It has also been shown that patients suffering from anxiety show enhanced amygdala activation to emotional face stimuli when compared to controls and that the degree of activation correlates with the severity of their pathology (Etkin and Wager, 2007; Stein et al., 2007). Further, high anxiety has been shown to be associated with increased amygdala volume and connectivity (Qin et al., 2014). Animal models have also been used to show the role of the amygdala in anxiety-related behaviors (Davis, 1992; Roozendaal et al., 2009). Concordant with human data, amygdala lesions in animals reduce anxiety-related behaviors (Amaral, 2002; Kalin et al., 2004). Lastly, recent optogenetic manipulations of the amygdala were shown to have an acute effect on anxiety-related behaviors (Tye et al., 2011; Felix-Ortiz et al., 2013; Kim et al., 2013a).

Along with its role in anxiety, the amygdala's role in social behavior has also been well-established (Brown and Schafer, 1888; Klüver and Bucy, 1937; Jonason and Enloe, 1971; Kling and Steklis, 1976; Amaral et al., 2003; Adolphs, 2010). Diseases such as autism, Urbach-Wiethe disease, Kluver-Bucy syndrome, and Williams syndrome have provided clues regarding the involvement of the amygdala in social behavior as amygdala damage or dysfunction appears to precipitate aberrant sociality in these diseases (Baron-Cohen et al., 2000; Meyer-Lindenberg et al., 2005; Todd and Anderson, 2009; Adolphs, 2010; Haas et al., 2010). In support of the notion that the amygdala plays a role in social functioning, it has also been found that higher amygdala volume and stronger intrinsic connectivity is correlated with having a larger, more complex social network (Bickart et al., 2011, 2012). In animals, amygdala lesions also result in changes in social behavior (Rosvold et al., 1954; Emery et al., 2001; Amaral et al., 2003; Machado and Bachevalier, 2006; Machado et al., 2008; Adolphs, 2010; Bliss-Moreau et al., 2013). These studies strongly suggest that amygdala circuitry may be involved in mediating aspects of anxiety and social function and that pathology in these domains may arise from a common, aberrant pathway involving the amygdala.

## Optogenetic investigation of the amygdala and hippocampus

The amygdala is composed of functionally and anatomically distinct subnuclei that include the basolateral amygdala (BLA) and the central amygdala (CeA) (McDonald, 1982a,b; Pape and Pare, 2010). The CeA can be further subdivided into medial (CeM) and lateral (CeL) subnuclei (McDonald, 1982a,b; Sah et al., 2003). Selective manipulation of these various subnuclei using optogenetics has revealed that specific regions have distinct roles in controlling behavior (Ciocchi et al., 2010; Haubensak et al., 2010). For example, Ciocchi and colleagues demonstrated that CeM activation as well as CeL inhibition caused unconditioned freezing, an innate behavioral fear response in rodents (Ciocchi et al., 2010). Amygdala subnuclei not only have distinct functions, they are also intricately connected. The BLA is glutamatergic and sends projections to the CeA, a GABAergic nucleus (Paré and Smith, 1993; Pape and Pare, 2010). Within the CeA, the CeL sends inhibitory projections to the main output nucleus of the amygdala, the CeM (Krettek and Price, 1978). Optogenetics allows for projection-specific manipulations (Stuber et al., 2011; Tye et al., 2011; Tye and Deisseroth, 2012) and because of this technological advantage, researchers are able to examine the distinct role of specific circuits in governing different aspects of behavior. For example, Tye and colleagues showed that the BLA-CeL projections bidirectionally control anxiety-related behaviors (Tye et al., 2011). Interestingly, manipulations of this specific circuit had the opposite effect to manipulations of the BLA as a whole (Tye et al., 2011). This study established the importance of using optogenetics to dissect the functional roles of specific projections.

The amygdala is connected to a number of downstream and upstream regions that may be candidates for circuitry involved in anxiety and social behavior (Davis, 1992; McDonald, 1998; Fendt and Fanselow, 1999; Davidson, 2002). One region of interest is the ventral hippocampus (vHPC) due to its robust and reciprocal connections with the amygdala (O'Donnell and Grace, 1995; Pikkarainen et al., 1999; Chen and Etkin, 2013) and its involvement in both anxiety and social behavior. In humans, the hippocampus and amygdala have been shown to be dependent on one another during the encoding of emotional memories (Richardson et al., 2004). Further, amygdala:hippocampal volume ratio corresponds to the severity of anxiety observed in some patients (MacMillan et al., 2003). In addition to these clues in the human literature, previous work in rodents has shown that the vHPC is important for the expression of fear- and anxiety-related behaviors (Kjelstrup et al., 2002; Bannerman et al., 2004; Kheirbek et al., 2013). Along with its role in anxiety, the vHPC is also involved in social behaviors. In non-human primates, hippocampal lesions lead to abnormal responses to social signals and degradation of social bonds (Machado and Bachevalier, 2006). Experiments using social interaction paradigms in rodents also provided evidence that the vHPC is involved in social behavior (Cadogan et al., 1994; Deacon et al., 2002; McHugh et al., 2004).

## The BLA-vHPC circuit facilitates anxiety-related behaviors and impairs social interaction

Taken together, the studies discussed above strongly suggest that the amygdala and the vHPC are both involved in mediating anxiety-related behaviors; however, the contribution of the specific connection between these two regions has been poorly understood. As demonstrated by the optogenetic manipulation of specific projections from the amygdala (Tye et al., 2011), specific projections from a brain region may encode information that cannot be gleaned from non-specific activation or inhibition of an entire brain region. Given that the non-specific activation of the BLA was anxiogenic while activation of BLA-CeL was anxiolytic, one possible explanation was that the majority of BLA neurons projected to other regions and mediated an anxiogenic phenotype. Indeed, the BLA projects to many other regions implicated in anxiety, including the medial prefrontal cortex (mPFC), bed nucleus of the stria terminalis (BNST) and the vHPC (Bishop, 2007; Etkin and Wager, 2007; Kim et al., 2013a).

**Figure 2:**
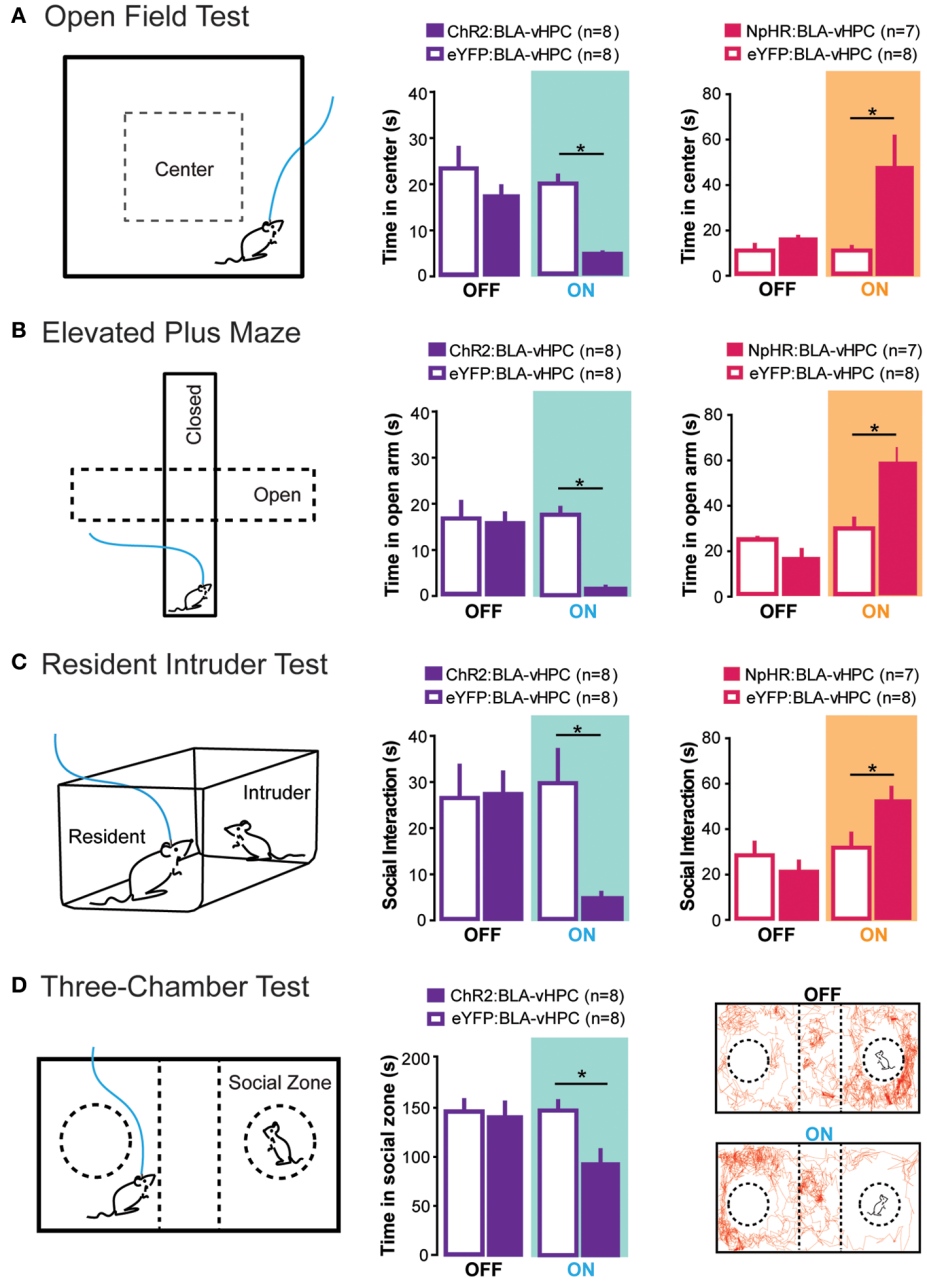
**Projections from the BLA to the vHPC bidirectionally modulate anxiety-related behaviors as well as social interaction**. To perform all of the following experiments, channelrhodopsin-2 or an enhanced version of halorhodopsin was injected into the basolateral amygdala (BLA) and an optical fiber was placed above the ventral hippocampus (vHPC). This allowed selective optogenetic manipulation of BLA projections to the vHPC. **(A)** In the open field test, decreased time spent in the center was measured as a proxy for increased anxiety. Excitation of BLA-vHPC projections lead to an increase in anxiety-related behaviors while inhibition of BLA-vHPC projections lead to a decrease in anxiety-related behaviors. **(B)** In the elevated plus maze, decreased time in the open arms was measured as a proxy for increased anxiety. Excitation of BLA-vHPC projections lead to an increase in anxiety-related behaviors while inhibition of BLA-vHPC projections lead to a decrease in anxiety-related behaviors. **(C)** The resident intruder test was used to measure social interaction. The overall score of social interaction included body sniffing, anogenital sniffing, direct contact, and close following (<1 cm). Activation of BLA-vHPC projections led to a decrease in social interaction while inhibition of BLA-vHPC projections led to an increase in social interaction. **(D)** The three-chamber test was used as a test for sociability. Time spent in the social zone was measured as proxy for sociability. Activation of BLA-vHPC projections lead to a decrease in time spent in the social zone. **(A–D)** These experiments demonstrate that anxiety and social interaction can be modulated at the level of the neural circuit. Additionally activation of BLA-vHPC projections leads to increases in anxiety-related behaviors as well as decreases in social interaction while inhibition of the same circuit causes a decrease in anxiety-related behaviors as well as an increase in social interaction. Data are mean values and error bars represent SEM. For the Open Field Test and Elevated Plus Maze, the light-off epochs were pooled and averaged (One Way ANOVA with Bonferroni post test, ^*^p < 0.01). For the Resident Intruder Test and the Three-Chamber Test statistics described in Felix-Ortiz and Tye (2014) (^*^p < 0.05).

To study the BLA-vHPC circuit, light-sensitive opsins were expressed in glutamatergic BLA projection neurons and an optical fiber was positioned above BLA axon terminals within the vHPC for precise illumination. In line with hypotheses that posit amygdala hyperactivity underlies anxiety (Anagnostaras et al., 1999; Drevets, 2003; Kalin and Sheltona, 2003; Carter and Krug, 2009), *in vivo* phasic light activation of ChR2-expressing BLA terminals within the vHPC transiently and significantly increased anxiety-related behaviors in the OFT, EPM, and novelty-suppressed feeding paradigm (Figure 2). These light-evoked anxiogenic effects were prevented by intra-vHPC glutamate antagonism, demonstrating that excitatory projections from the BLA to the vHPC are sufficient to mediate anxiety. Conversely, *in vivo* light inhibition of BLA axons in the vHPC reduced anxiety-related behaviors in the OFT and EPM (Figure 2) (Felix-Ortiz et al., 2013). Therefore, this study identified the BLA-vHPC as a circuit that bidirectionally governs anxiety-related behaviors in a manner opposite to that of the BLA-CeL circuit (Tye et al., 2011). Together, these data show that BLA projections to different downstream targets can have opposing roles in modulating anxiety-related behavior.

The evidence that anxiety is correlated with deficits in social interaction and that both the amygdala and hippocampus appear to be involved in both processes, prompted investigation into the role of the BLA-vHPC circuit in social behavior. Using the approach described above to target BLA terminals in the vHPC, the effects of BLA-vHPC manipulations on rodent sociability were tested during two behavioral paradigms: the juvenile-intruder test and the three-chamber sociability test. Inhibition of BLA-vHPC projections increased sociability in these two paradigms, while excitation of this pathway decreased sociability (Figure 2) (Felix-Ortiz and Tye, 2014). Just as in the previous study, intra-vHPC glutamate receptor antagonism attenuated the effects of optogenetic stimulation demonstrating that glutamatergic transmission from the BLA to the vHPC was critical for mediating the light-induced changes in social behavior. This study provides evidence that although complex social behaviors likely involve a distributed neural network across multiple brain regions (File and Seth, 2003), social interaction can still be modulated by manipulations of a single circuit element.

Combined, these two studies reveal that the BLA-vHPC circuit can control anxiety and social interaction in the same manner that would be predicted by the animal and human literature discussed throughout this review. This demonstrates that anxiety and social behaviors can be mechanistically linked at the level of the neural circuit. These studies also provide evidence that the co-morbidity of anxiety disorders and autism spectrum disorders (Gillott et al., 2001; Pine et al., 2008; MacNeil et al., 2009) may, in part, be caused by dysfunction of circuits that mediate both anxiety and social behavior.

## Conclusion

The above-mentioned studies provide the first evidence that a common circuit can link both social behavior and anxiety. Interestingly, these studies also provide evidence that manipulation of a single population of synapses can effectively change social behavior, which is likely dependent on multiple circuits acting in concert (Baron-Cohen et al., 2000; File and Seth, 2003; Bachevalier and Loveland, 2006; Rushworth et al., 2013). Perhaps the manipulation of BLA-vHPC drives multiple downstream circuits modulating these complex behaviors, or otherwise alters the transmission in a corticolimbic loop that maintains behavioral states. It remains unclear from these studies whether the observed changes in social behavior seen after optogenetic manipulations of BLA-vHPC are due to direct control of social behavior or are secondary to the changes in the state of anxiety.

The fact that social deficits and general anxiety do not always co-occur suggests that the mechanism underlying these two forms of behavior are dissociable (Gonzalez et al., 1996; Schneier, 2006; Liu and Smith, 2009; Toth et al., 2012). It is possible that the difference between the variable social deficits seen in general anxiety and the socially-specific anxiety seen in social anxiety disorder arises from differences in functional connectivity between circuits governing both behaviors. Lastly, the high co-morbidity of anxiety disorders with autism spectrum disorders suggests that these diseases may share common pathological mechanisms (De Bruin et al., 2007; Simonoff et al., 2008). There are mouse models of autism that show co-expression of social deficits and general anxiety-related behaviors, while other models show social deficits without changes in general anxiety-related behaviors (Moy et al., 2008). Elucidating the circuit differences between various models may provide insight as to why some autistic patients have co-morbid anxiety disorders while others do not.

Future studies should aim to differentiate between non-overlapping circuits by identifying specific circuit elements wherein manipulation causes changes in either social behavior assays or anxiety-related behaviors without altering the other. Toward this end, optogenetically stimulating molecularly defined neurons within specific circuits could further elucidate where the separation and overlap lies between circuits controlling social behavior and anxiety. An example of this was seen in recent work which provided evidence that dopaminergic projections from the ventral tegmental area to the nucleus accumbens modulates social behavior, while dopaminergic projections to the mPFC modulates anxiety with no effect on social behavior (Gunaydin et al., 2014). Indeed, using optogenetics to clarify how circuits governing social behaviors interact with circuits governing other complex behaviors will likely provide insight about the mechanism by which social function is impaired in a wide array of psychiatric diseases.

### Conflict of interest statement

The authors declare that the research was conducted in the absence of any commercial or financial relationships that could be construed as a potential conflict of interest.
